# Preparation of Monoacylglycerol Derivatives from Indonesian Edible Oil and Their Antimicrobial Assay against *Staphylococcus aureus* and *Escherichia coli*

**DOI:** 10.1038/s41598-019-47373-4

**Published:** 2019-07-29

**Authors:** Jumina Jumina, Wenggi Lavendi, Tubagus Singgih, Sugeng Triono, Yehezkiel Steven Kurniawan, Mamoru Koketsu

**Affiliations:** 1 Department of Chemistry, Faculty of Mathematics and Natural Sciences, Universitas Gadjah Mada, Yogyakarta, 55281 Indonesia; 2 Department of Chemistry and Biomolecular Science, Faculty of Engineering, Gifu University, Gifu, 501-1112 Japan

**Keywords:** Bacteriology, Synthetic chemistry methodology

## Abstract

In the present work, linoleic acid and oleic acid were isolated from Indonesian corn oil and palm oil and they were used to prepare monoacylglycerol derivatives as the antibacterial agent. Indonesian corn oil contains 57.74% linoleic acid, 19.88% palmitic acid, 11.84% oleic acid and 3.02% stearic acid. While Indonesian palm oil contains 44.72% oleic acid, 39.28% palmitic acid, 4.56% stearic acid and 1.54% myristic acid. The oleic acid was purified by using Urea Inclusion Complex (UIC) method and its purity was significantly increased from 44.72% to 94.71%. Meanwhile, with the UIC method, the purity of ethyl linoleate was increased from 57.74% to 72.14%. 1-Monolinolein and 2-monoolein compounds were synthesized via two-step process from the isolated linoleic acid and oleic acid, respectively. The preliminary antibacterial assay shows that the 1-monolinolein did not give any antibacterial activity against *Staphylococcus aureus* and *Escherichia coli*, while 2-monoolein showed weak antibacterial activity against *Staphylococcus aureus*.

## Introduction

Indonesia is a mega biodiversity country because it is located near the equator line, therefore, many kinds of plant oil are commercially available^1^. Among them, corn oil and palm oil are important in daily life because they contain a high amount of monounsaturated fatty acid and polyunsaturated fatty acid^2,3^. They have been widely used as pharmaceuticals, cosmetic, and food material^4–6^. Corn oil mainly contains linoleic acid (56.3%), oleic acid (30.1%), palmitic acid (8.1%) and stearic acid (2.6%)^7^. Meanwhile, palm oil mainly contains palmitic acid (44.0%), oleic acid (39.2%), linoleic acid (10.1%), stearic acid (4.5%) and myristic acid (1.1%)^8,9^.

On the other hand, recently, a great concern is on the food sustainability field to ensure food safety and quality for human health^10^. One of the factors which affect the safety and quality of the food material is its resistance against bacterial activities^11^. A lot of organic compounds were synthesized or isolated from the natural products and evaluated for their antimicrobial activities. However, they have serious drawbacks, such as high price, limited natural product resources, complicated synthesis route, low security for human health, low physical and chemical stability, *etc*.^12,13^.

Several researchers investigated the antimicrobial activity of fatty acid derivatives. However, they show poor antimicrobial activity against *Escherichia coli* (*E. coli*) and *Staphylococcus aureus* (*S. aureus*)^14–17^. In general, esterification reaction of fatty acid with glycerol increased the antibacterial activity^18,19^. In our previous work, the 1-monolaurin compound was prepared in a simple and efficient method from lauric acid and glycerol in the presence of the *p*-toluenesulfonic acid as the catalyst^20,21^. Monolaurin exhibits high antibacterial activity against gram-positive bacteria such as *S. aureus*, either in planktonic and biofilm mode of growth^12^. Monolaurin derivative was able to decrease the minimum inhibition concentration (MIC = 125 μM) for its antibacterial activity against *S. aureus* compared with lauric acid (MIC = 250–500 μM). It was due to the ability of monolaurin to incorporate on the *S. aureus* bacterial lipid bilayer through the formation of spherical protrusions within 2 min^22^. Galbraith *et al*. reported that lauric acid is the most potential gram-positive antibacterial agent among the saturated fatty acids, while linoleic acid is the most potential gram-positive antibacterial agent among the unsaturated fatty acids^23^. Yoon *et al*. also reported that oleic acid shows antibacterial activity against *S. aureus* through damaging the bacterial cell membrane^24^. Even though the monolaurin derivative has been deeply investigated, the investigation for the other monoacylglycerol is still limited. Since Indonesian corn oil and palm oil are rich with linoleic acid and oleic acid content, it is interesting to evaluate monolinolein and monoolein antibacterial activity against *S. aureus* and *E. coli*.

In the present study, 1-monolinolein and 2-monoolein were prepared from commercially available Indonesian corn oil and palm oil, and their antibacterial activity was investigated against *E. coli* and *S. aureus*. First, the linoleic acid and oleic acid were isolated through hydrolysis reaction of triglycerides and then purified by using UIC method. Afterwards, each of them was used to produce monoacylglycerol compound. Their antibacterial activity was evaluated against *E. coli* and *S. aureus* through well-diffusion method. The effect of the alkyl chain towards its antimicrobial activity is discussed.

## Materials and Methods

### Materials

The used Indonesian edible oil in this work is commercial palm oil “Bimoli” and commercial corn oil “Mozella”. The other chemicals such as urea, potassium hydroxide, sulfuric acid, Amberlyst-15 and *Thermomyces Lanuginosa* as Lipase enzyme were purchased in pro analytic grade and used without further purification. The synthesis of 1,2-*O*-isopropylidene glycerol has been carried out in a similar procedure to the previously described^25^.

### Isolation of fatty acids from plant oil

In this work, corn oil was used as the linoleic acid source, while palm oil was used as the oleic acid source. Plant oil (25 g) was mixed with 11% wt/v of potassium hydroxide in ethanol (50 mL). The mixture was refluxed for 90 min and then *n*-hexane (35 mL) was added into the mixture and the aqueous phase was isolated. The aqueous phase was acidified with 1 M (M = mol dm^−3^) of sulfuric acid until the pH of the aqueous phase reached 1.0. The generated organic phase was dried over anhydrous sodium sulfate and then preconcentrated through evaporation. The obtained fatty acids, either from commercial corn oil or palm oil, were characterized by FT-IR (Fourier Transform Infrared, Shimadzu Prestige 21).

Fatty acid: 19.49 g of yellow liquid product from corn oil. 20.14 g of yellow liquid product from palm oil. FT-IR (NaCl cell, cm^−1^): 3356 (broadened, -OH), 2916 (C-H stretching), 1697 (C=O carboxylic acid), 1458 (CH_2_ bending), 1049 (C-O carboxylic acid).

The obtained of fatty acids (18 g) were esterified by using 1% wt/v sulfuric acid solution in alcohol (22 mL) and the mixture was refluxed for 90 min. Methanol was used as the alcohol for fatty acids from the palm oil, while ethanol was used as the alcohol from the corn oil. After the reaction, the mixture was extracted with *n*-hexane (12 mL) and then neutralized with 10% wt/v sodium bicarbonate solution in water (20 mL). The organic phase was separated and then dried over anhydrous sodium sulfate. The obtained fatty acid alkyl esters were characterized by FT-IR and GC-MS (Gas Chromatography-Mass Spectrometry, Shimadzu QP 2010S with Agilent GC Type 6890-MS Type 5973).

Fatty acid alkyl ester: FT-IR (NaCl cell, cm^−1^): 2924 (C-H stretching), 1743 (C=O ester), 1458 (CH_2_ bending), 1180 (C-O ester).

5.86 g of fatty acid methyl ester from palm oil. GC: 1.54% methyl myristate (retention time (t_R_) = 26.5 min, M^+^ = 242), 39.28% methyl palmitate (t_R_ = 30.9 min, M^+^ = 270), 44.72% methyl oleate (t_R_ = 34.2 min, M^+^ = 296), 4.56% methyl stearate (t_R_ = 34.5 min, M^+^ = 298).

15.31 g of fatty acid ethyl ester from corn oil. GC: 19.88% ethyl palmitate (t_R_ = 42.3 min, M^+^ = 284), 57.74% ethyl linoleate (t_R_ = 45.8 min, M^+^ = 308), 11.84% ethyl oleate (t_R_ = 46.1 min, M^+^ = 310), 3.02% ethyl stearate (t_R_ = 46.3 min, M^+^ = 312).

### Isolation of Ethyl linoleate by using Urea Inclusion Complex (UIC) method

Isolation and preconcentration of ethyl linoleate from the fatty acid ethyl esters derived from corn oil were carried out in a similar manner to the method which has been reported by Hai-bo *et al*.^26^. Briefly, fatty acid ethyl esters (27.2 g) were added into 25% wt/v urea solution in ethanol (160 mL) and stirred quickly. The nitrogen gas was bubbled to preconcentrate the mixture. Then the mixture was stored in the refrigerator for 24 h. The formed crystals were filtered, and the filtrate was acidified until the pH of the aqueous phase reached 1.0. Afterwards, the ethyl linoleate was extracted with *n*-hexane (40 mL). The organic phase was separated and concentrated by using nitrogen gas flow. The ethyl linoleate as an isolated product was characterized by GC-MS, ^1^H- and ^13^C-NMR (Nuclear Magnetic Resonance, 500 MHz, JEOL JNM ECZ500R/S1).

Ethyl linoleate: 11.36 g of yellow liquid product. FT-IR (NaCl cell, cm^−1^): 2924 (C-H stretching), 1736 (C=O ester), 1458 (CH_2_ bending), 1180 (C-O ester). GC: 1.77% ethyl palmitate (t_R_ = 26.2 min, M^+^ = 284), 24.36% ethyl oleate (t_R_ = 33.1 min, M^+^ = 310), 72.14% ethyl linoleate (t_R_ = 35.2 min, M^+^ = 308). ^1^H-NMR (500 MHz, CDCl_3_): δ 0.89 (t, 3H, -CH_3_), 1.25 (t, 3H, -O-CH_2_-C**H**_**3**_), 1.30 (m, 16H, -CH_2_-), 2.04 (m, 4H, -C**H**_**2**_-CH=), 2.78 (t, 2H, -C**H**_**2**_-CO), 2.83 (t, 2H, =CH-C**H**_**2**_-CH=), 4.13 (q, 2H, -O-CH_2_-), 5.35 (m, 4H, -C**H**=C**H**-). ^13^C-NMR (125 MHz, CDCl_3_): δ 14.28 (-CH_3_), 22.64 (-**C**H_2_-CH_3_), 25.02 (-**C**H_2_-CH_2_-CO), 25.67 (=CH-**C**H_2_-CH=), 27.25–31.59 (other -CH_2_-), 34.39 (-**C**H_2_-CO), 60.19 (-O-**C**H_2_-), 128.08 & 130.19 (-**C**H=**C**H-), 173.91 (-COO-).

### Synthesis of 1-monolinolein compound

This process was carried out in the two-step process, *i.e*. synthesis of the 1,2-*O*-isopropylidene glycerol linoleate compound and deprotection by using Amberlyst-15 as solid acid catalyst. First, ethyl linoleate (6.2 g) was reacted with 1,2-*O*-isopropylidene glycerol (16 g) and sodium carbonate (2.2 g) at 413 K for 30 h. The mixture was washed with distilled water until neutral. Then *n*-hexane (40 mL) was added into the mixture to extract the desired product. The organic phase was dried over anhydrous sodium sulfate and the solvent was evaporated.

The isopropylidene glycerol linoleate (3.9 g) as the intermediate product, and Amberlyst-15 (0.6 g) were dissolved in ethanol (20 mL), and the mixture was stirred at 300 K for 72 h. The residue was filtered and the product was extracted with dichloromethane (20 mL). The organic phase was washed with distilled water until neutral and then dried over anhydrous sodium sulfate. The organic phase was evaporated to obtain 1-monolinolein as the final product. The final product was characterized by LC-MS, ^1^H- and ^13^C-NMR.

1-monolinolein: 2.88 g as yellow liquid product. FT-IR (NaCl cell, cm^−1^): 3379 (broadened, OH), 2924 (C-H stretching), 1728 (C=O ester), 1443 (CH_2_ bending), 1180 (C-O ester). GC: 41.30% cis-monolinolein (t_R_ = 32.3 min, M^+^ = 262), 10.63% 1-monoolein (t_R_ = 32.5 min, M^+^ = 265), 41.93% trans-monolinolein (t_R_ = 41.3 min, M^+^ = 262). ^1^H-NMR (500 MHz, CDCl_3_): δ 0.89 (t, 3H, -CH_3_), 1.30 (m, 14H, -CH_2_-), 1.63 (m, 2H, -C**H**_**2**_-CH_2_-CO), 2.04 (m, 4H, -C**H**_**2**_-CH=), 2.79 (t, 2H, =CH-C**H**_**2**_-CH=), 3.62 & 3.69 (s, 2H, OH), 3.80 (dod, 2H, -C**H**_**2**_-OH), 3.93 (q, 1H, -COOC**H**-), 4.19 (dod, 2H, -C**H**_**2**_-OOC-), 5.34–5.35 (m, 4H, -C**H**=C**H**-). ^13^C-NMR (125 MHz, CDCl_3_): δ 14.13 (-CH_3_), 22.59 (-**C**H_2_-CH_3_), 24.89 (-**C**H_2_-CH_2_-CO), 25.63 (=HC-**C**H_2_-CH=), 27.20 (=HC-**C**H_2_-), 29.12–31.53 (other -CH_2_-), 34.14 (-**C**H_2_-CO), 63.30 (-CH_2_-OH), 65.08 (-CH_2_-O-), 70.19 (-CH-OH), 128.04 & 130.24 (-**C**H=**C**H-), 174.34 (-COO-).

### Isolation of oleic acid by using UIC method

Isolation and preconcentration of methyl oleate from the fatty acid methyl esters derived from palm oil were carried out in a similar manner to the method which has been reported by Hai-bo *et al*.^26^. Fatty acid methyl esters (6.3 g) were added into 25% wt/v urea solution in methanol (50 mL) and the mixture was stirred quickly. The nitrogen gas was bubbled to preconcentrate the mixture. Then the mixture was stored in the refrigerator for 24 h. The formed crystals were filtered and the filtrate was acidified until the pH of the aqueous phase reached 1.0. Afterwards, the methyl oleate was extracted with *n*-hexane (40 mL). The organic phase was separated and concentrated by using nitrogen gas flow. The methyl oleate as an isolated product was characterized by GC-MS.

Methyl oleate: 0.8 g of product. GC: 5.29% methyl palmitate (t_R_ = 30.7 min, M^+^ = 270), 94.71% methyl oleate (t_R_ = 34.2 min, M^+^ = 296).

To obtain oleic acid, the methyl oleate (0.8 g) was hydrolyzed with 7% wt/v potassium hydroxide in ethanol (15 mL). The mixture was refluxed for 90 min and then *n*-hexane (15 mL) was added into the mixture. The aqueous phase was separated and acidified until the pH of the aqueous phase reached 1.0. The organic phase was formed after the acidification process was dried over anhydrous sodium sulfate and the organic phase was evaporated. The oleic acid as the product was characterized by FT-IR.

Oleic acid: 0.1 g as yellow liquid product. FT-IR (NaCl cell, cm^−1^): 3009 (broadened, OH), 2924 (C-H stretching), 1705 (C=O carboxylic acid), 1458 (CH_2_ bending), 1049 (C-O carboxylic acid).

### Synthesis of 2-monoolein compound

This process was carried out in two steps, *i.e*. synthesis of the triolein compound and selective hydrolysis process by using Lipase enzyme. At first, oleic acid (13 g) was reacted with glycerol (0.7 g) and 1 M of sulfuric acid (0.5 mL) at 383 K for 5 h. The mixture was neutralized by a small addition of 1 M sodium hydroxide solution. The mixture was extracted by using *n*-hexane (45 mL) to extract the triolein compound. The organic phase was dried over anhydrous sodium sulfate and the solvent was evaporated. The triolein product was characterized by using FT-IR, GC-MS, ^1^H- and ^13^C-NMR.

Triolein: 6.04 g as yellow liquid product. FT-IR (NaCl cell, cm^−1^): 2924 (C-H stretching), 1713 (C=O ester), 1458 (CH_2_ bending), 1173 (C-O ester). ^1^H-NMR (500 MHz, CD_3_OD): δ 0.86 (t, 9H, -CH_3_), 1.28 (m, 60H, -CH_2_-), 1.58 (m, 6H, -C**H**_**2**_-CH_2_-CO), 1.99 (m, 12H, -C**H**_**2**_-CH=CH-C**H**_**2**_-), 2.29 (t, 6H, -C**H**_**2**_-CO), 4.10–4.29 (dod, 4H, -COOC**H**_**2**_-), 5.26 (m, 1H, -COOC**H**-), 5.32 (m, 3H, -C**H**=C**H**-). ^13^C-NMR (125 MHz, CD_3_OD): δ 14.32 (-CH_3_), 22.89 (-**C**H_2_-CH_3_), 25.01 (-**C**H_2_-CH_2_-CH_3_), 25.33 (-**C**H_2_-CH_2_-CO), 27.43 (-**C**H_2_-CH=CH-**C**H_2_-), 29.3–34.41 (other -CH_2_-), 35.2 (-**C**H_2_-CO), 62.32 (-COO**C**H_2_-), 69.10 (-COO**C**H-), 129.93 & 130.22 (-**C**H=**C**H-), 173.09 & 173.50 & 180.36 (-COO-).

Triolein (1.9 g) was dissolved in the mixture of *n*-hexane (15 mL) and water (1.6 mL). *Thermomyces Lanuginosa* (0.3 g) was added into the solution, and the mixture was incubated at 160 rpm and 310 K for 24 h. After the incubation process, the desired product was extracted with dichloromethane (10 mL). The organic phase was neutralized with 5% wt/v sodium hydroxide solution then dried over anhydrous sodium sulfate. The organic phase was evaporated to obtain 2-monoolein as the final product. The final product was characterized by LC-MS (Liquid Chromatography-Mass Spectrometry, Acquity HPLC-SQD MassLynx v4–1 SCN 805), ^1^H- and ^13^C-NMR.

2-monoolein: 0.58 g as yellow liquid product. LC: single peak (t_R_ = 3.01 min, M^+^ = 356.06). ^1^H-NMR (500 MHz, CD_3_OD): δ 0.89 (t, 3H, -CH_3_), 1.32 (m, 20H, -CH_2_-), 1.58 (m, 2H, -C**H**_**2**_-CH_2_-CO), 2.02 (m, 4H, -C**H**_**2**_-CH=CH-C**H**_**2**_-), 2.14 (t, 2H, -C**H**_**2**_-CO), 3.52–3.54 (dod, 4H, -C**H**_**2**_-OH), 4.11 (m, 1H, -COOC**H**-), 5.01 (s, 2H, OH), 5.33 (m, 2H, -C**H**=C**H**-). ^13^C-NMR (125 MHz, CD_3_OD): δ 14.61 (-CH_3_), 23.85 (-**C**H_2_-CH_3_), 27.94 (-**C**H_2_-CH_2_-CO), 28.21 & 28.29 (-**C**H_2_-CH=CH-**C**H_2_-), 30.41–33.02 (other -CH_2_-), 33.16 (-**C**H_2_-CO), 33.73 (-**C**H_2_-OH), 39.45 (-**C**H-OH), 130.92 & 131.03 (-**C**H=**C**H-), 183.4 (-COO-).

### Antimicrobial assay against *S. aureus* and *E. coli*

The antimicrobial assay of the synthesized products was carried out at Microbiology Laboratory of Faculty of Veterinary Medicine, Universitas Gadjah Mada by using a well-diffusion method as previously reported^25,27^. *S. aureus* FNCC 0047 was used as a representative of gram-positive bacteria while *E. coli* FNCC 0091 was used as a representative of gram-negative bacteria. The 100 μg/mL chloramphenicol was used as the positive control and 20.0% polyethylene glycol 400 (PEG 400) in distilled water was used as the negative control. Briefly, brain heart broth solution in distilled water (6.5% w/v) was sterilized at 394 K under 15 psi pressure for 15 min. Then the bacteria were separately introduced into the prepared media at a different petri dish. The synthesized product was dissolved in 20.0% PEG 400 at the desired concentration and the sample (50 μL) was injected to the prepared well with a fixed diameter at 8.0 mm. The inhibition zone was calculated from the formed transparent zone due to the antibacterial activity of the sample.

## Results and Discussion

### Isolation of fatty acids from plant oil

**Figure 1:**
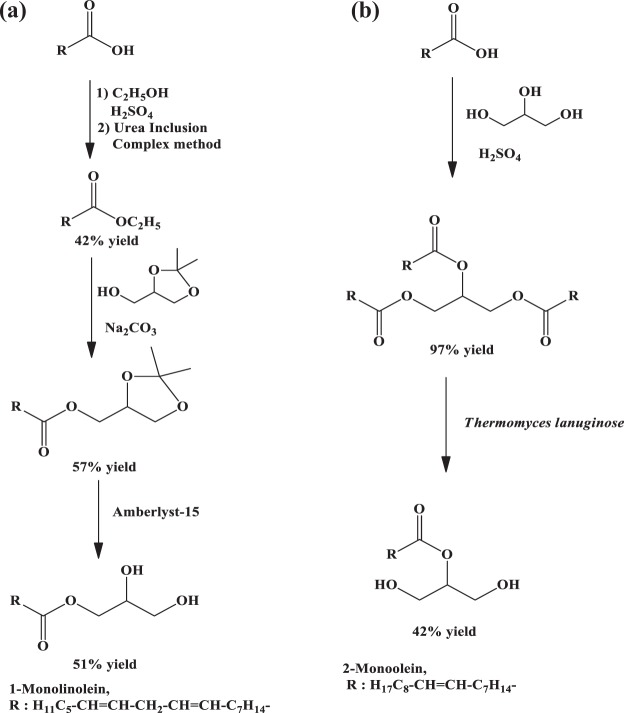
Synthesis scheme of (**a**) 1-monolinolein and (**b**) 2-monoolein compounds.

In this work, monoacylglycerol derivatives were synthesized and the reaction scheme is shown in Fig. 1. Initially, fatty acids were isolated from Indonesian palm oil and corn oil resources via hydrolysis reaction. The triglycerides were hydrolyzed under alkaline condition and the fatty acids were isolated after acidification. The presence of the carboxylic acid group was confirmed from the OH (broad, 3356 cm^−1^) and C=O (1697 cm^−1^) peaks on the FTIR spectra. Afterwards, the fatty acids were esterified either with methanol or ethanol to form fatty acid methyl ester and fatty acid ethyl ester, respectively. This process was carried out in order to analyze fatty acid composition from the Indonesian palm oil and corn oil by using GC-MS. It was found that Indonesian palm oil “Bimoli” contains 44.72% oleic acid, 39.28% palmitic acid, 4.56% stearic acid and 1.54% myristic acid. Meanwhile, corn oil “Mozela” contains 57.74% linoleic acid, 19.88% palmitic acid, 11.84% oleic acid and 3.02% stearic acid. These results are slightly different from other previous literature, due to the different geographical and climate factors^7,8^.

### Isolation of Oleic acid by using UIC method

**Figure 2:**
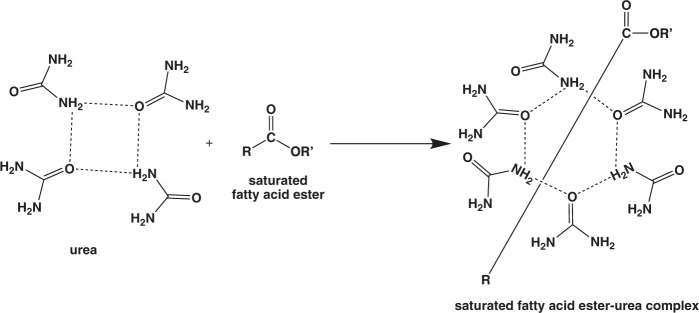
Selective urea complexation reaction with saturated fatty acid ester from fatty acid esters mixture. Straight line R-COOR’ representative the saturated alkyl chain of fatty acid ester.

**Figure 3:**
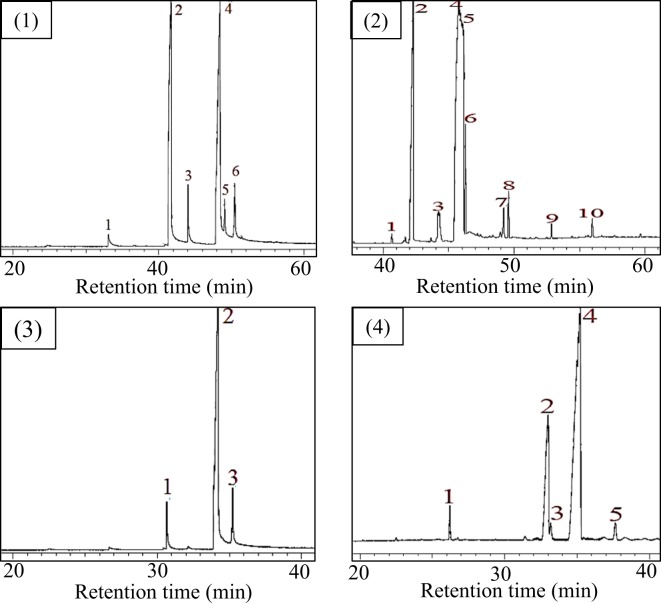
GC chromatogram of (1) isolated fatty acid esters from palm oil before UIC purification method, (2) isolated fatty acid esters from corn oil before UIC purification method, (3) isolated fatty acid esters from palm oil after UIC purification method, (4) isolated fatty acid esters from corn oil after UIC purification method.

UIC method is one of the purification methods which is based on the selective crystallization between urea and saturated fatty acids^26,28^. The selective interaction between urea and the saturated fatty acids was occurred due to the suitable intermolecular interaction. Smith reported that urea formed tetragonal crystal at inner diameter of 0.57 nm. However, after urea complexation with the saturated fatty acid, the hexagonal crystal was formed at a larger inner diameter (0.8–1.2 nm) depends on the fatty acid structure^29^. The schematic reaction equation is shown in Fig. 2. The limited space on the urea crystal is believed as the main reason why the bent structure of the unsaturated fatty acids could not be incorporated. Because of that, the saturated fatty acids were crystallized at low temperature while the unsaturated fatty acids remained in the filtrate solution. Therefore, a simple separation of saturated and unsaturated fatty acids can be achieved. Figure 3 shows GC chromatograms before and after UIC purification of fatty acid esters derived from palm oil and corn oil. For palm oil, methyl myristate and methyl stearate were eliminated from the mixture after UIC method, thus the purity of methyl oleate was significantly increased from 44.72% to 94.71%. Meanwhile, the purity of ethyl linoleate was increased from 57.74% to 72.14%, which is remarkable. This is because the urea could not interact either with ethyl linoleate or ethyl oleate in the sample, therefore, the purity of ethyl linoleate is less than 73%. However, these preliminary findings are useful to preconcentrate the unsaturated fatty acids from the commercial sample in a simple procedure.

### Synthesis of 1-monolinolein and 1-monoolein compounds

The synthesis of 1-monolinolein was carried out through synthesis of isopropylidene glycerol linoleate and followed by deprotection using a solid acid catalyst. While the 2-monoolein was prepared through synthesis of the triolein compound and selective hydrolysis process using Lipase enzyme. The different methods existed in order to selectively obtain 1-monoacyl and 2-monoacyl derivatives. The protection of glycerol with acetone will leave one unreacted alcohol which can be further reacted through transesterification reaction, thus it is suitable enough to produce the 1-monolinolein product. Meanwhile, *Thermomyces Lanuginosa* enzyme could selectively hydrolyze 1,3-diester compound^30,31^, thus it is used to obtain 2-monoolein.

Chemical structure of the intermediates and final products was confirmed through spectrometry analysis. The presence of the hydroxyl peak for 2-monoolein and 1-monolinolein was confirmed from a broadened peak on the FT-IR spectra. The ^1^H-NMR spectra confirmed two hydroxyl protons on each product at 3.62 and 3.69 ppm for 1-monolinolein (Supplementary Fig. S1). While the equivalent hydroxyl protons from 2-monoolein were confirmed from ^1^H-NMR peak at 5.33 ppm as a singlet (Supplementary Fig. S2). Moreover, the ^13^C-NMR spectra confirmed a single carbonyl ester at 174.34 and 183.4 ppm for 1-monolinolein and 2-monoolein products, respectively (Supplementary Figs S3 and S4).

### Preliminary antibacterial assay against *S. aureus* and *E. coli*

The antibacterial assay of the synthesized products was carried out by using a well-diffusion method with 100 μg/mL chloramphenicol as the positive control and 20.0% PEG 400 as the negative control. The result of the antibacterial assay is listed in Table 1. It was found that 1-monolinolein did not show any antibacterial activity against *S. aureus* and *E. coli*, while 2-monoolein showed weak antibacterial activity against *S. aureus*. Either ethyl linoleate or ethyl oleate did not give any antibacterial activity against *S. aureus* and *E. coli* at 100 mg/mL concentration. Even though ethyl linoleate and ethyl oleate were used in the present work, their antibacterial assay results are similar to their corresponding fatty acids, i.e. linoleic acid and oleic acid, respectively. In the previous report, Galbraith *et al*. reported that linoleic acid gave no antibacterial activity against gram-negative bacteria (*E. coli*) which is in agreement with the previous result^23^. However, Galbraith *et al*. did not investigate the antibacterial activity of linoleic acid against *S. aureus*. Yoon *et al*. reported that oleic acid gave antibacterial activity against *S. aureus* 18Z^24^. Since the used *S. aureus* in the present work is *S. aureus* FNCC 0047, the different antibacterial activity may due to either the difference of the used *S. aureus* bacteria strain or lower oleic acid concentration used in the present work.

**Table 1:** Antimicrobial assays of monoacylglycerol products against *S. aureus* and *E. coli*.

Product	Inhibition zone (cm)		Reference
	*S.aureus*	*E. coli*	
1-Monolinolein^a^	—	—	This work
2-Monoolein^a^	1.20	—	This work
Ethyl linoleate^a^	—	—	This work
Ethyl oleate^a^	—	—	This work
1-Monoolein^a^	1.10	0.85	^32^
1-Monocaprin^a^	2.50	1.00	^33^
1-Monolaurin^a^	1.58	1.27	^21^
2-Monolaurin^a^	—	—	^34^
1-Monomyristin^a^	0.91	—	^25^
2-Monomyristin^a^	1.10	0.50	^25^
2-Monopalmitin^a^	—	—	^25^
Negative control^b^	—	—	
Positive control^c^	8.10	2.60	

^a^100 mg/mL concentration. ^d^20.0% PEG 400 was used as the negative control. ^e^100 μg/mL chloramphenicol was used as the positive control.

Debois *et al*. stated that antibacterial activity of fatty acid derivatives was influenced by the shape and the structure of the compounds, such as length of alkyl chain, and number, position and orientation of double bonds^19^. From Table 1, it was found that 1-monoolein exhibit higher antibacterial activity than 1-monolinolein against *S. aureus* and *E. coli*. In this case, the difference between antibacterial activity of 1-monolinolein and 1-monoolein is due to the effect of the number of double bonds. The number of double bonds for the 1-monoolein (only one double bond) is less than the 1-monolinolein (two double bonds exist), therefore the structure of 1-monoolein is more linear and well-arranged than the 1-monolinolein. Because of that, 1-monoolein destabilize bacterial cell membrane and increase its permeability, therefore, exhibits higher antibacterial activity. However, 2-monoolein gave a similar antibacterial activity against *S. aureus* but no antibacterial activity against *E. coli*. When the oleyl substituent was located in the edge of the glycerol, the interaction between the fatty acid chain with the gram-negative bacterial cell wall seemed to be strong enough to exhibit antibacterial activity. Yoon *et al*. reported that the suitable interaction between monoacylglycerol compounds and bacterial cell membrane caused bacterial cell membrane destabilization by increasing cell permeability. Because of that destabilization, there are two consequences, i.e. bacteriostatic action (inhibition of bacterial cell growth) and bactericidal action (bacterial cell was lysis)^24^.

From Table 1 monoacylglycerol derivatives exhibit higher antibacterial activity of monoacylglycerol derivatives against *S. aureus* as a representative of gram-positive bacteria than *E. coli* as a representative of gram-negative bacteria, which is in agreement with many reports^19,24^. The order of antibacterial activity against *S. aureus* is 1-monocaprin > 1-monolaurin > 2-monoolein > 1-monoolein, 2-monomyristin > 1-monomyristin > 1-monolinolein, 2-monolaurin, 2-monopalmitin. While the order of the antibacterial activity against *E. coli* is 1-monolaurin > 1-monocaprin > 1-monoolein > 2-monomyristin > 1-monolinolein, 2-monoolein, 2-monolaurin, 1-monomyristin, 2-monopalmitin. Weaker antibacterial activity of monoacylglycerol derivatives against *E. coli* than *S. aureus* was found because gram-negative bacteria (*E. coli*) are protected by the outer membrane while this component is absent in gram-positive bacteria (*S. aureus*)^19^. The order of antibacterial activity of monoacylglycerol against *S. aureus* is consistence with the length of the alkyl chain. Shorter alkyl chain of monoacylglycerol generally gave higher antibacterial activity^24^. However, the antibacterial activity against *S. aureus* of 1-monoolein and 2-monoolein is higher than 2-monomyristin and 2-monolaurin although alkyl chain of oleic acid is longer than either myristic acid or lauric acid. It is probably due to the presence of double bond on oleic acid which contributes to the antibacterial activity as reported before^19^. Meanwhile, the order of antibacterial activity of monoacylglycerol against *E. coli* is consistency with the length of alkyl chain, except for 1-monolaurin because 1-monolaurin was well-known for its high antibacterial activity. It is because 1-monolaurin is able to destabilize the bacterial cell membrane by increasing cell permeability as well as disrupt the electron transport chain of bacteria^24^.

## Conclusions

We reported a success purification of linoleic acid and oleic acid from Indonesian corn oil and palm oil, respectively. The purity of oleic acid was remarkably increased from 44.72% to 94.71% by using UIC method. Meanwhile, the purity of ethyl linoleate was increased from 57.74% to 72.14% because the urea can interact either with ethyl linoleate or ethyl oleate in the mixture. The 1-monolinolein and 2-monoolein compounds were successfully prepared from the isolated linoleic acid and oleic acid by two-step process, and their chemical structure was confirmed from FTIR, MS, ^1^H-NMR and ^13^C-NMR spectra. The 1-monolinolein did not give any antibacterial activity against *S. aureus* and *E. coli*, while 2-monoolein showed weak antibacterial activity against *S. aureus*.

## Supplementary information

Preparation of Monoacylglycerol Derivatives from Indonesian Edible Oil and Their Antimicrobial Assay against Staphylococcus aureus and Escherichia coli

## Data Availability

The datasets generated during and/or analyzed during the current study are available from the corresponding author on reasonable request.
