# Supplementary material for: Preparation of Monoacylglycerol Derivatives from Indonesian Edible Oil and Their Antimicrobial Assay against Staphylococcus aureus and Escherichia coli
Source: Sci Rep. 2019 Jul 29;9:10941. doi: 10.1038/s41598-019-47373-4 (PMC6662904; doi:10.1038/s41598-019-47373-4)
Supplement: Supplementary file 1 — Preparation of Monoacylglycerol Derivatives from Indonesian Edible Oil and Their Antimicrobial Assay against Staphylococcus aureus and Escherichia coli [file 41598_2019_47373_MOESM1_ESM.pdf]

Supplementary Information

Preparation of Monoacylglycerol Derivatives from Indonesian Edible Oil and Their Antimicrobial Assay against *Staphylococcus aureus* and *Escherichia coli*

Jumina Jumina[1,*], Wenggi Lavendi[1], Tubagus Singgih[1], Sugeng Triono[1], Yehezkiel Steven Kurniawan[1] and Mamoru Koketsu [2]

[1]Department of Chemistry, Faculty of Mathematics and Natural Sciences, Universitas Gadjah Mada, Yogyakarta 55281, Indonesia

[2]Department of Chemistry and Biomolecular Science, Faculty of Engineering, Gifu University, Gifu 501-1112, Japan

*Corresponding author: jumina@ugm.ac.id Tel.: +62 274 545188

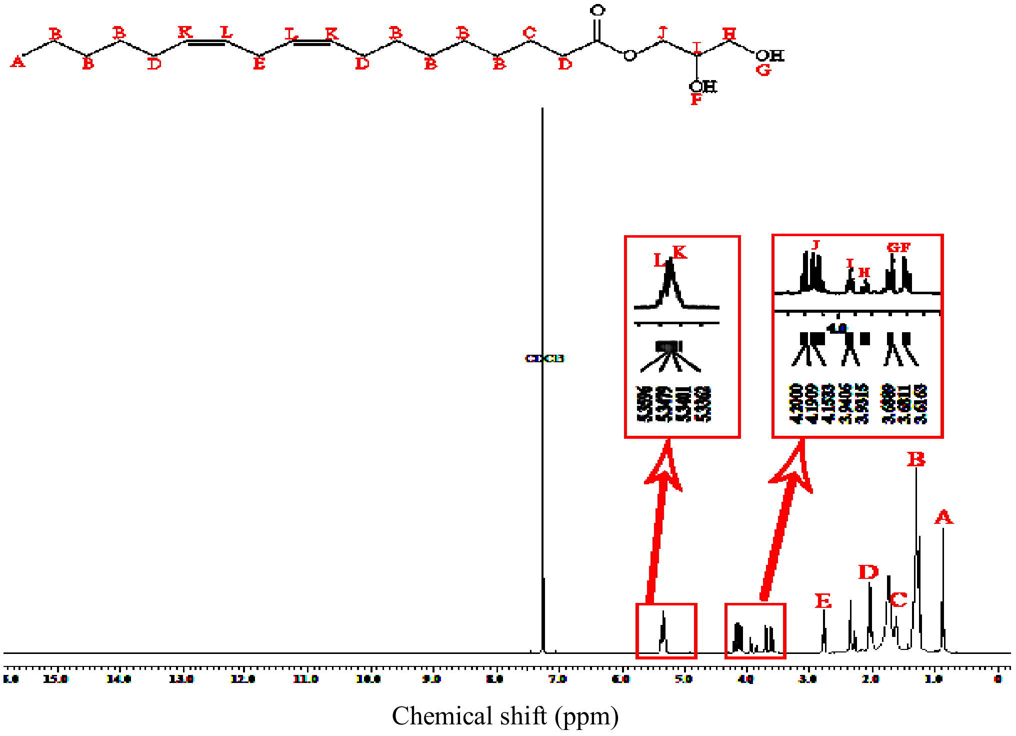

Figure S1. ^{1}H-NMR spectra of 1-monolinolein compound

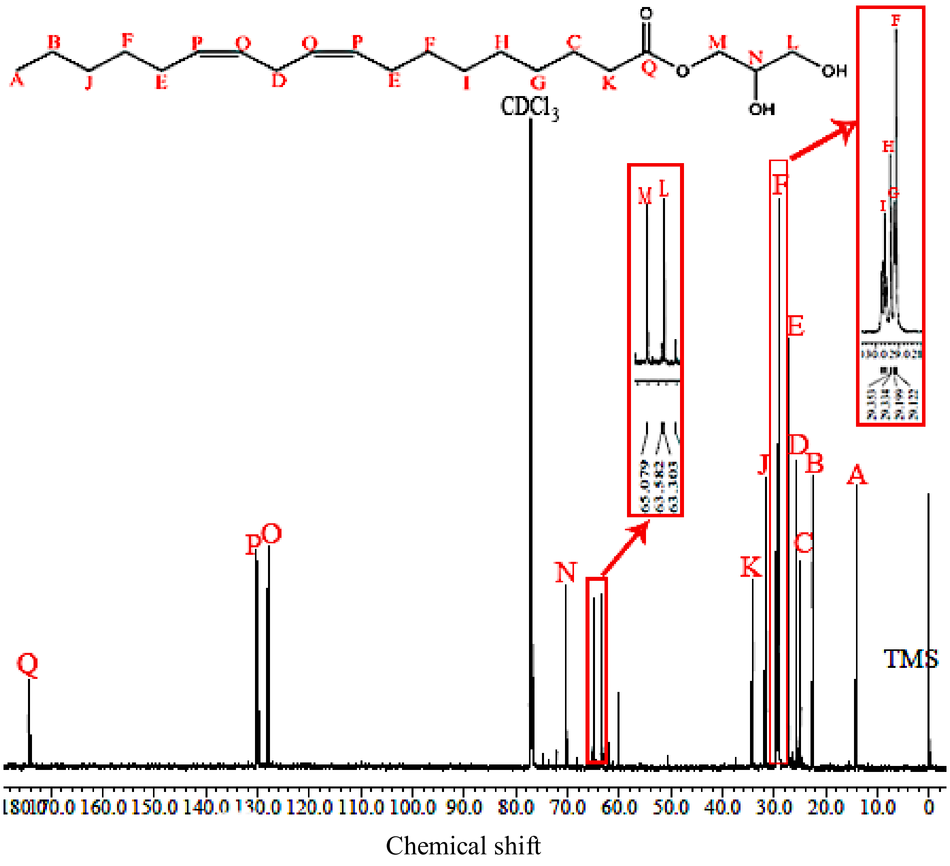

Figure S2. ^{13}C-NMR spectra of 1-monolinolein compound

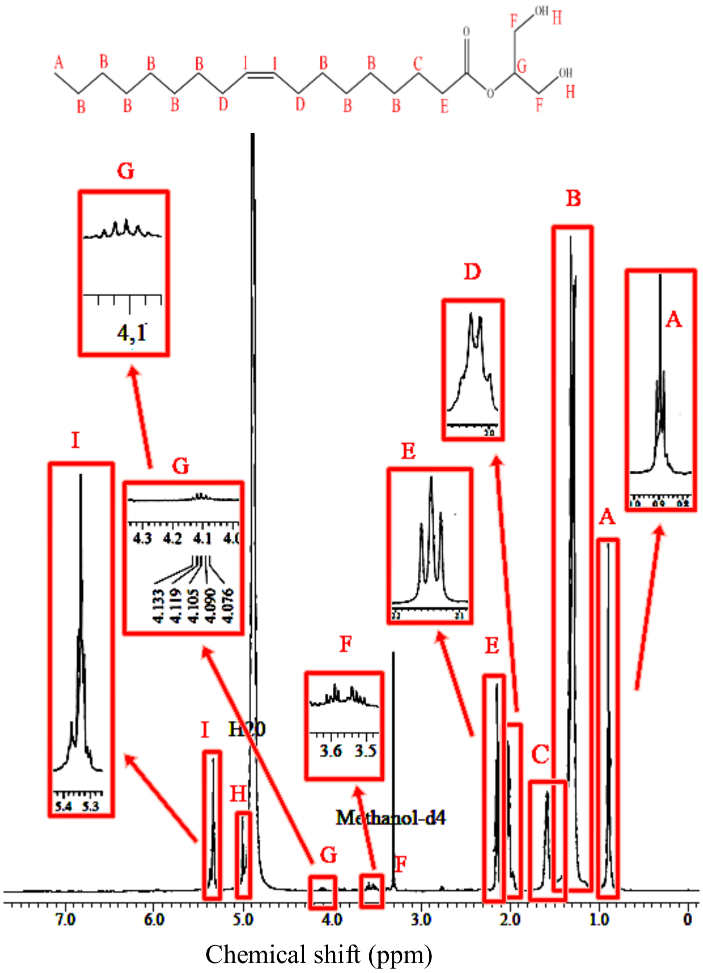

Figure S3. ^{1}H-NMR spectra of 2-monoolein compound

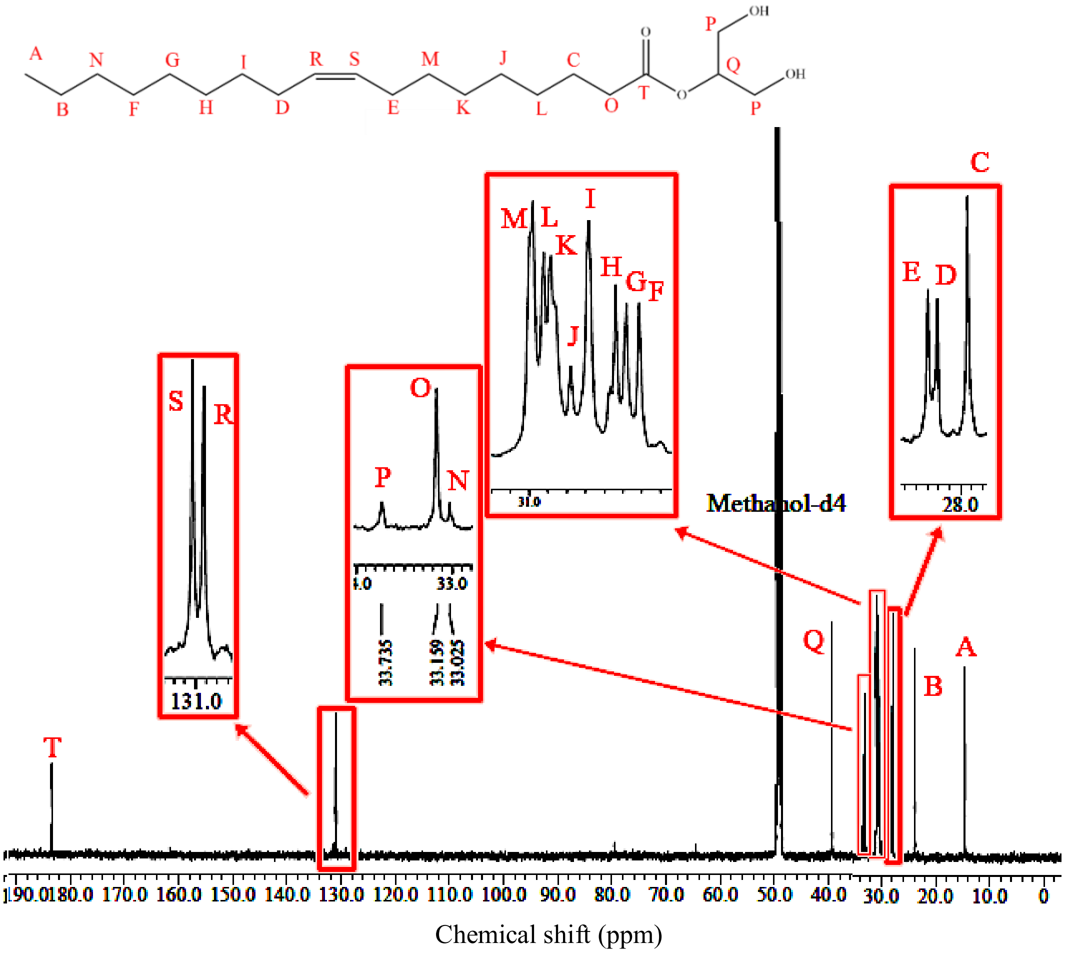

Figure S4. ^{13}C-NMR spectra of 2-monoolein compound
